# Factors associated with tuberculosis treatment delay in patients co-infected with HIV in a high prevalence area in Brazil

**DOI:** 10.1371/journal.pone.0195409

**Published:** 2018-04-06

**Authors:** Betânia M. F. Nogueira, Valéria C. Rolla, Kevan M. Akrami, Susan M. Kiene

**Affiliations:** 1 Division of Epidemiology and Biostatistics, San Diego State University, San Diego, California, United States of America; 2 Instituto de Pesquisa Clínica Evandro Chagas, Fundação Oswaldo Cruz, Rio de Janeiro, Brazil; 3 Division of Infectious Diseases, University of California San Diego, La Jolla, California, United States of America; Indian Institute of Technology Delhi, INDIA

## Abstract

**Background:**

Worldwide, about 11% of Tuberculosis (TB) cases occur in people living with HIV (PLHIV) and it is the leading cause of death in this population. An important step towards reducing the incidence and mortality of TB in PLHIV is to reduce the time from onset of symptoms to treatment. Factors related to TB treatment delay therefore need to be understood.

**Methods:**

Using data from a prospective cohort study of patients diagnosed with TB at the National Institute of Infectious Disease, at the Oswaldo Cruz Foundation, Rio de Janeiro, Brazil we conducted a survival analysis to identify factors associated with patient and health care treatment delay. In our analysis we included patients who were co-infected with TB and HIV (n = 201). Patients were followed during the course of their TB treatment and information regarding duration of symptoms, sociodemographics and clinical characteristics were collected at the baseline visit.

**Results:**

The median time from onset of initial symptoms to prescription of TB treatment (total delay) was 82 days. From initiation of symptoms to first visit at INI clinic (patient delay), the median was 51 days. From first visit to initiation of treatment (health care delay) the median was 16 days. Illiteracy was associated with greater patient delay [Hazard Ratio (HR) = 2.25, CI 95% 1.29–3.94]. Having had a previous episode of TB (HR = 0.53, CI 95% 0.37–0.74) and being married (HR = 0.71, CI 95% 0.54–0.94) were inversely related to patient delay. Illiteracy was also associated with greater health care delay (HR = 2.83, CI 95% 1.25–5.47) in contrast to high viral load (HR = 0.37, CI 95% 0.24–0.54) and weight loss greater than 10% (HR = 0.54, CI 95% 0.37–0.8), both of which were inversely related to health care delay.

**Conclusions:**

This study highlights the existence of factors that lead to greater risk of delayed treatment of TB among patients co-infected with HIV and TB. These include factors that can be assessed through targeted interventions which have implications for improving treatment outcomes and, through reduced duration of infectiousness, reduce the incidence of TB in Brazil.

## Introduction

Despite significant advances in the treatment of HIV and tuberculosis (TB) worldwide, both HIV and TB remain major public health issues, and are of particular concern when presenting together as co-infections. Globally, about 11% of TB cases occur in people living with HIV (PLHIV) and it is the leading cause of death in this population. [1–3] Co-infection with HIV/TB is also the leading cause of hospitalization of adults and children living with HIV.[4]

As the most populated country of Latin America, Brazil accounts for more than 40% of all new HIV infections in the region, with an estimated prevalence of 0.4–0.6%. In 2015 there were estimated to be 830,000 PLHIV in Brazil, 44,000 new HIV infections, and 15,000 AIDS-related deaths. [4,5] The incidence rate of HIV was 19.1/100,000 population in Brazil in 2015, though nearly twice as high in the state of Rio de Janeiro. [5]

Compounded with this HIV epidemic, Brazil ranks 16^th^ among the 22 countries with the highest TB burden in the world. Rio de Janeiro state ranks second in TB incidence (61.2/100,000) and has the highest mortality rate (5.0/100,000) in the country. In 2015, Rio was also among the states with a high post-mortem diagnosis of TB, which is one of the indicators of late detection of TB cases. In 2015, 11.23% of confirmed TB cases in Rio were among PLHIV. [6]

Prevention and treatment of TB among PLHIV is a priority for both HIV/AIDS and TB programs. One of the main steps toward the control of this co-infection is diagnosing and treating TB cases early, as delay in treatment increases the risk of death, [7,8] morbidity,[9,10] prolonged hospitalization, [10,11] and promotes transmission in the community.[12,13] In a prior paper from the same cohort as the present study, Schmaltz et al(8) found that TB treatment delay was associated with early mortality in patients co-infected with HIV/TB who were followed at the Oswaldo Cruz Foundation (FIOCRUZ), Rio de Janeiro. However, the study did not examine factors associated with treatment delay.

In the literature TB delay is often divided in patient delay–the time from onset of symptoms to until the patients see the first health care facility; health care system delay–the time from first health care seeking for diagnosis until the initiation of treatment; and total delay—the time from onset of symptoms to initiation of treatment. [14]

Studies from multiple countries have identified various factors associated with delay in TB diagnosis and treatment, with significant variation. Factors differ according to social and cultural issues, local TB and HIV prevalence, and characteristics of public health systems. The majority of these studies focused on TB mono-infection with few focused exclusively on those co-infected with HIV. [15–18] In Brazil, there have been studies examining factors associated with TB treatment delay [15–19] but only Coimbra et al [15] focused on PLHIV. The study evaluated total treatment delay in patients with only pulmonary TB who were attending HIV care services at the time that they developed TB symptoms. The presence of systemic symptoms, asthenia, chest pain, use of illicit drugs and a smear negative sputum were all associated with greater total delay in their study. [15] To the best of our knowledge, no study to date has included TB-HIV co-infected patients with recent diagnosis of HIV, nor separately examined patient and health care system delay in Brazil.

Given the diversity in culture, populations and distribution of health care, the drivers of delayed TB treatment may be very different even within Brazil. Through a better understanding of these drivers, particularly in areas with higher prevalence of TB, interventions can be designed to improve control of TB in PLHIV and align with the WHO goals to reduce TB prevalence and mortality. [20,21] The current study examined factors associated with TB treatment delay in HIV patients in an area with high TB and HIV burden in Brazil.

## Materials and methods

### Population and design

The data used in this study were drawn from a prospective cohort study carried out at the National Institute of Infectious Disease (INI), at the Oswaldo Cruz Foundation (FIOCRUZ). FIOCRUZ, under the Ministry of Health, is the main institution of science and technology in health in Brazil. INI is a reference unit for infectious diseases at the FIOCRUZ campus in Rio de Janeiro that, among other infectious diseases, cares for TB and HIV patients in integrated programs. Participants in the parent study were followed at the Clinical Research laboratory on Mycobacteria (LAPCLIN-TB), which provides care to patients referred to INI from the public healthcare network, private hospitals, and clinics. The parent study began in 2000 and is ongoing, enrolling approximately 100 patients per year. As of January 2016, 1,600 patients have been enrolled and followed up for 6 months after initiating TB treatment. Inclusion criteria for the parent study are: ≥18 years of age, diagnosed with pleuropulmonary, extra-pulmonary or disseminated TB, and giving written informed consent to participate. The diagnosis of TB was confirmed by a positive acid fast bacilli smear, Gene Xpert or culture from clinical specimens. In cases without bacteriological confirmation, the diagnosis was established by histopathological examination, together with clinical, epidemiological and radiological findings consistent with TB. Exclusion criteria are: having a positive culture for a non-TB mycobacteria, lack of therapeutic response in those without microbiological confirmation and the identification of an alternative diagnosis explaining the symptoms.

Our analyses included all patients co-infected with TB-HIV, who entered the study between 1/01/2008 and 01/07/2016. Patients with bone, ocular or skin TB were excluded, since these clinical forms can have very subtle, asymptomatic presentations, making it difficult to be compared to the other forms. Those with multi-drug resistant (MDR) TB were also excluded since MDR TB is rare in Brazil (1.5% among all the new TB cases in 2016) [22] and in previous years patients were referred elsewhere. Finally, patients who were referred to the TB clinic after they had initiated their TB treatment were excluded for the purposes our study. Patients presenting with other opportunistic diseases were not excluded as long as the participant had a concomitant TB diagnosis.

Participants provided written informed consent at the date of the first prescription of TB specific therapy, which was considered the baseline visit. The study was approved by the INI Institutional Review Board. At baseline, patients were interviewed by INI research physicians and underwent relevant exams and laboratory tests (including HIV testing).

### Data collection

Using a structured data collection instrument at the baseline visit, study physicians interviewed patients and collected their: gender, age, monthly income, education in years, marriage status (married/not married), housing status (homeless or not), past or present history of incarceration, history of psychiatric disease, amount of weight loss since the beginning of symptoms (greater than 10% weight loss since the onset of symptoms), duration of symptoms associated with TB, clinical presentation of TB, history of previous episodes of TB, and co-morbidities, such as diabetes, chronic obstructive pulmonary disease, hepatitis B and C, opportunistic diseases, amongst others.

The duration of symptoms related to TB was collected by asking the patient the date of onset of one of the following symptoms: fever, cough, night sweats, chills, dyspnea, weight loss, chest pain, anorexia, malaise, purulent sputum, hemoptysis, abdominal pain, diarrhea, lymphadenopathy. Other less suggestive symptoms were included if later identified as associated with the TB episode (i.e. headache as a symptom of TB meningitis).

The physicians also collected information about current use of illicit drugs and alcohol (Y/N to each) during the baseline interview. Potential problematic alcohol use was assessed with the CAGE questionnaire, with scores of 2 or greater indicating clinically significant alcohol problems. Smoking status was recorded as current/former/non-smoker. All participants who reported to be currently daily or less than daily cigarette smokers were considered “current smokers”. Former daily or less than daily cigarette smokers who reported completely quitting any time in the past were considered “former smokers”. “Non-smokers” are those who reported never smoking. Other variables of interest collected at the baseline visit included time from the first positive HIV serology and current use of antiretrovirals (Y/N). The most recent results of the CD4 cell count and HIV viral load from the preceding four months were also recorded at the baseline visit. Hemoglobin, Albumin, Hepatitis B and C tests, AFB smear, culture, Gene Xpert and histopathological examinations were collected as clinically indicated. Gene Xpert became available in 2014, thus only a few participants had results for this test for TB.

#### Microbiologic methods

Ziehl-Neelsen staining was used for the detection of acid-fast bacilli in sputum smears and biopsy specimens. Sputum and biopsy samples were cultured in Löwenstein-Jensen medium. Culture of blood specimens used the lysis-centrifugation method. GeneXpert MTB/RIF assay was performed in sputum samples of some participants enrolled from 2014 to 2016.

We examined two outcomes: patient delay and health care delay. We defined patient delay as the interval between the onset of symptoms and presentation to INI, health care delay as the interval between the date of first visit to INI and the initiation of TB treatment, and total delay as the sum of both intervals. The outcome variables were continuous (number of days). For participants with a previous diagnosis of HIV followed at the INI HIV clinic, the first visit for the purpose of the study was the first visit that the patient complained of a symptom of concerning for TB to the HIV clinician. For the participants newly diagnosed with HIV we defined “first visit” as the first visit to INI due to symptoms associated with TB.

### Statistical analysis

We performed analyses using SAS version 9.5 and conducted bivariate analyses using Log-rank tests to examine the relationship between each factor of interest and the two outcome variables. Factors with p<0.25 in bivariate analysis were included in the multivariable model and kept in the model if they had p<0.10. The variable “clinical form of TB” (pleuropulmonary, extrapulmonary or miliary/disseminated) and “education level” were included and kept in both multivariate models regardless of p-value, given the importance of the clinical form of TB in treatment delay, as previously demonstrated in literature.[23–25]

We conducted a survival analysis using the Weibull proportional hazard model. We compared the risk of TB diagnosis across 2 or more groups using Hazard Rates (HR). Considering that all the participants met the event in both analysis (first consultation at the clinic and TB treatment initiation), the focus was on the time component of the survival analysis. HR and confidence intervals for the risk of diagnosis of TB were calculated, using 95% confidence. A HR greater than 1 means that the risk of reaching the outcome is increasing with time. In other words, it represents the risk of having the first clinic visit later or initiating TB treatment later. Conversely, HR smaller than 1 represents the risk of reaching the outcomes earlier.

Some variables in the dataset were missing data: 8 (4%) for the variable “Homeless”(Y/N), 22 (10.9%) for baseline CD4, 17 (8%) for baseline viral load and 23 (11.4%) for baseline albumin. The missing laboratory results were only in individuals newly diagnosed with HIV. Missing data followed a non-random pattern. We used multiple imputation to create 20 imputed datasets and performed the final analysis with a pooled dataset. From the initial cohort a total of 24 participants were excluded from the analysis: 5 did not have HIV status, 2 had bone TB, 1 had ocular TB and 2 had skin TB. 14 patients had already started treatment by the time they were admitted to the TB clinic.

## Results

A total number of 201 patients diagnosed with TB-HIV were included in the analysis. The mean age was 39 years, ranging from 19 to 67 years. Most participants resided in Rio de Janeiro (66.17%) and most were male (70%). About 43% were diagnosed with HIV during the TB investigation and 57% knew of their diagnosis of HIV before the TB episode. In the previously diagnosed HIV patients, the mean time from HIV diagnosis to first visit to the clinic was 93 months. In this group, 73% of individuals were using antiretroviral therapy (ART), but only 27% of those were virologically suppressed (viral Load < 40 copies/mL).

The median time of total delay was 82 days. The median patient delay was 51 days and the median health care delay was 16 days. Among the group with previous HIV diagnosis the median total delay, patient delay and health care system delay were 82.5 (range 7–532), 32.5 (0–409) and 22 (range 0–367), respectively. Among those with recent diagnosis of HIV the median delays were 92.5 (range 7–532), 70.5 (8–394), and 13.5 (range 0–400) respectively. Tables 1 and 2 provide detailed sociodemographic and clinical characteristics of the population studied and descriptive statistics for the additional potential predictor variables considered in the analysis.

**Table 1:** Baseline characteristics of study participants (n = 201).

Variable	Frequency	Percent	Variable	Frequency	Percent
*SOCIODEMOGRAPHICS*			*CLINICAL CHARACTERISTICS*		
**City of residency**			**Past History of TB**		
Rio de Janeiro	133	66.17	Yes	37	18.4
Other	68	33.83	No	164	81.6
**Gender**			**Clinical form of TB**		
Female	60	30	Pleuro-pulmonary	116	57.7
Male	141	70	Extra-pulmonary	26	12.9
**Sexual behavior**			Miliary/disseminated	59	26.4
Heterosexual	128	64	**Weight loss greater than 10%**		
Homosexual/bisexual	73	36	Yes	142	70.6
**Homeless**			No	59	29.4
Yes	7	3.5	**Did Gene Xpert**		
No	194	96.5	Yes	33	16.4
**Self-referred ethnicity**			No	168	83.6
White	80	39.8	**Gene Xpert result**		
Black/brown	121	60.2	Positive	23	11.4
**Education level**			Negative	10	5
Illiterate	12	6	Was not done	168	83.6
Up to 8 years	92	45.8	**Acid- Fast Bacilli (AFB) smear result**		
More than 8 years	97	48.3	Positive	93	46.3
Monthly income per household*			Negative	107	53.2
< 0.5	120	59.7	Was not done	1	0.5
0.5–1.49	66	32.8	**Culture**		
> = 1.5	15	7.5	Positive	142	70.6
**Marriage status**			Negative	49	24.4
Married	63	31.34	Was not done	10	5
Non married	138	68.66	**Admitted to a hospital during diagnostic investigation**		
**Smoking**			Yes	67	33.3
Current smokers	60	29.9	No	134	66.7
Former smokers	44	21.9	**Had diagnosis of HIV prior to this episode of TB**		
Non smokers	97	48.3	Yes	115	57.2
**Alcohol abuse classified as CAGE> = 2**			No	86	42.8
Yes	135	67.2	Use of ART**		
No	66	32.8	Yes	84	41.8
**Current use of illicit drugs**			No	31	15.4
Yes	57	28.35	Status of HIV treatment**		
No	144	71.65	NAÏVE	31	27
**Current use of cocaine**			On ART (VL not suppressed)	61	53
Yes	40	20	On ART (VL suppressed)	23	20
No	161	80	**Comorbidities**		
**Current use of crack**			Yes	83	41.3
Yes	12	6	No	118	58.7
No	189	94	**Hepatitis B**		
**Current use of marijuana**			Yes	8	4
Yes	29	16	No	193	96
No	169	84	**Hepatitis C**		
**Current use of other drugs**			Yes	13	6.5
Yes	6	3	No	188	93.5
No	195	97	**Diabetes**		
			**Yes**	10	5
			**No**	191	95

* In Brazilian minimal wages

** Among patients with previous diagnosis of HIV.

**Table 2:** Descriptive statistics of baseline characteristics of study participants.

Variables	N	Mean	Std Dev	Median	Range	Interquartile range
Symptoms to admission (days)	201	73.85	78.46	51	0–409	69
Admission to treatment (days)	201	38.24	59.06	16	0–380	41
Symptoms to treatment (days)	201	112.08	99.83	82	7–704	89
Age (years)	201	38.58	10.22	37	19–67	15
Albumin (g/dL)	201	2.83	0.68	2.83	1–5.80	1
Hemoglobin (g/dL)	201	10.8	2.06	10.9	4.2–15.80	2.80
Cd4 (cells/mm^3^)	201	231.66	211	201	1–1242	263.50
Viral Load (IU/mL)	201	186,658.76	482,842.62	62,701	<40 -6,100,475	202,388
Time since past TB (years)	37	5.50	5.59	4	30-Jan	0
Time from diagnosis of HIV (months)*	115	92.9	63.3	81	1–268	67.60
Time of ARV use (months)*	84	70.67	57.44	59	1–235	66.30

* Among patients with previous diagnosis of HIV.

In the univariate analysis of factors associated with patient delay (Table 3), being illiterate was associated with increased risk of greater patient delay, while having previous diagnosis of HIV and previous history of TB was associated with a shorter patient delay. In the multivariate model, being married and having previous history of TB were associated with shorter patient delay, while being illiterate was associated with increased risk of greater patient delay. The HR, confidence intervals and p-values for the multivariate analysis are shown in Table 4.

**Table 3:** Univariate survival analysis of factors associated with patient delay** (n = 201).

Variable	*HR*	*95% CI*	*p-value*	Variable	*HR*	*95% CI*	*p-value*
**City**				**Comorbidities**			
Rio de Janeiro	1.15	0.87–1.52	0.32	Yes	1.08	0.83–1.42	0.56
Other (ref.)				No (ref.)			
**Gender**				**Hepatitis B**			
Male	1.11	0.83–1.48	0.47	Yes	0.60	0.44–1.68	0.65
Female (ref.)				No (ref.)			
**Age (years)**	1.00	0.99–1.01	0.80	**Hepatitis C**			
**Sexual behavior**				Yes	0.64	0.37–1.09	0.10
Heterosexual	1.05	0.79–1.38	0.75	No (ref.)			
Homosexual/bisexual (ref.				**Diabetes**			
**Ethnicity**				Yes	1.34	0.73–2.46	0.34
Black/brown	0.92	0.70–1.20	0.53	No (ref.)			
White (ref.)				**Weight loss greater than 10%**			
**Education level**				Yes	0.78	0.58–1.04	0.09
Illiterate	2.10	1.19–3.69	0.01*	No (ref.)			
Up to 8 years	1.06	0.81–1.38	0.68	**Clinical form**			
>8 years(ref.)				Pleuro-pulmonary	0.80	0.59–1.07	0.14
**Monthly income per person**				Extra-pulmonary	0.74	0.48–1.15	0.19
0–0.49	0.99	0.58–1.71	0.98	Disseminated/Miliary (ref.)			
0.5–1.49	2.59	0.44–1.37	0.37	**Albumin**	1.07	0.90–1.27	0.47
> = 1.5 (ref.)				**Hemoglobin**	0.96	0.91–1.02	0.22
**Homeless**				**Cd4 (100 cels/mL)**	0.99	0.93–1.06	0.81
Yes	0.68	0.32–1.47	0.33	**Viral Load (log10)**	0.71	0.86–1.06	0.42
No (ref.)				**Previous history of TB**			
**Marriage**				Yes	0.56	0.40–0.78	0.0005*
Yes	1.25	0.94–1.65	0.13	No (ref.)			
No (ref.)				**Previous diagnosis of HIV**			
**Imprisonment**				Yes	0.70	0.54–0.92	0.009*
Yes	0.93	0.62–1.39	0.72	No (ref.)			
No (ref.)				**Admission to a hospital during diagnostic investigation**				
**Smoking status**				Yes	1.01	0.76–1.34	0.95
Current Smoker	1.02	0.76–1.39	0.87	No (ref.)			
Former smoker	0.87	0.62–1.22	0.43				
Non- smoker (ref.)							
**Alcohol abuse**							
Yes	0.89	0.67–1.18	0.41				
No (ref.)							
**Illicit drugs**							
Yes	1.04	0.77–1.39	0.81				
No (ref.)							
**Cocaine**							
Yes	0.81	0.58–1.14	0.23				
No (ref.)							
**Crack**							
Yes	0.99	0.55–1.77	0.96				
No (ref.)							
**Marijuana**							
Yes	1.04	0.71–1.52	0.86				
No (ref.)							
**Other drugs**							
Yes	2.17	0.94–5.02	0.07				
No (ref.)							

* p<0.05

**Time from onset of symptoms to first visit to INI clinic.

**Table 4:** Univariate survival analysis of factors associated health care delay** (n = 201).

*Variable*	*HR*	*95% CI*	*p-value*	*Variable*	*HR*	*95% CI*	*p- value*
**City**				**Comorbidities**			
Rio de Janeiro	1.11	0.75–1.65	0.60	Yes	1.39	0.96–2.03	0.08
Other (ref.)				No (ref.)			
**Gender**				**Hepatitis B**			
Male	0.79	0.53–1.17	0.24	Yes	1.80	0.68–4.77	0.23
Female (ref.)				No (ref.)			
**Age (years)**	1.00	0.98–1.02	0.80	**Hepatitis C**			
**Sexual behavior**				Yes	0.54	0.26–1.14	0.11
Heterrosexual	1.24	0.84–1.82	0.27	No (ref.)			
Homosexual/bisexual (ref.)				**Diabetes**			
**Ethnicity**				Yes	2.90	1.11–7.55	0.03*
Black/brown	1.61	1.12–2.33	0.01*	No (ref.)			
White (ref.)				**Weight loss greater than 10%**			
**Education level**				Yes	0.66	0.45–0.99	0.04*
Illiterate	1.60	0.72–3.57	0.25	No (ref.)			
Up to 8 years	1.03	0.70–1.50	0.89	**Clinical form**			
>8 years (ref.)				Pleuro-pulmonary	1.38	0.90–2.09	0.14
**Monthly income (Brazilian minimal wage per person)**				Extra-pulmonary	1.26	0.69–2.32	0.45
0–0.49	1.86	0.80–3.32	0.15	Disseminated/Milliary (ref.)			
0.5–1.49	1.22	0.51–2.93	0.65	**Albumin**	1.19	0.89–1.59	0.24
> = 1.5 (ref.)				**Hemoglobin**	1.05	0.96–1.15	0.28
**Homeless**				**Cd4 (100 cels/mL)**	1.12	1.01–1.23	0.03*
Yes	1.85	0.58–0.88	0.30	**Viral Load (Log10)** 0.47		0.04–0.30	0.01*
No (ref.)				**Previous history of TB**			
**Marriage**				Yes	0.97	0.61–1.55	0.91
Yes	1.33	0.90–1.97	0.15	No (ref.)			
No (ref.)				**Previous diagnosis of HIV**			
**Smoking status**				Yes	1.49	1.02–2.17	0.04*
Current Smoker	0.87	0.57–1.33	0.52	No (ref.)			
Former smoker	0.98	0.61–1.59	0.95	**Admission to a hospital during diagnostic investigation**			
Non- smoker (ref.)				Yes	0.92	0.61–1.37	0.67
**Alcohol abuse**				Alcohol abuseNo (ref.)			
Yes	1.08	0.71–1.59	0.74	**Afb smear**			
No (ref.)				Positive	0.85	0.59–1.25	0.42
**Illicit drugs**				Negative (ref.)			
Yes	0.75	0.5–1.12	0.16	**Did Gene Xpert**			
No (ref.)				Yes	0.80	0.49–1.3	0.36
**Cocaine**				No (ref.)			
Yes	0.82	0.51–1.30	0.39	**Culture**			
No (ref.)				Positive	1.20	0.51–2.85	0.67
**Crack**				Negative	1.72	0.69–4.31	0.25
Yes	0.67	0.30–1.46	0.31	Not done (ref.)			
No (ref.)							
**Marijuana**							
Yes	0.61	0.37–1.02	0.06				
No (ref.)							
**Other drugs**							
Yes	0.75	0.24–2.34	0.62				
No (ref.)							

* p<0.05

** Time from first visit to clinic to initiation to initiation of TB therapy.

In the univariate analysis of factors associated with health care delay (Table 5), being black/brown, having diabetes, having higher CD4 counts and having previous diagnosis of HIV were associated with greater risk of greater health care delay. Having higher viral load and loss of more than 10% of bodyweight were associated with less health care delay. In the multivariate model, weight loss greater than 10% and higher viral load remained statistically significant factors inversely associated with health care delay. Being illiterate was found to be associated with longer health care delays. The HR, confidence intervals and p-values for the multivariate analysis are shown in Table 6.

**Table 5:** Multivariate survival analysis of factors associated with patient delay** (n = 201).

Variable	*HR*	*95% CI*	*p-value*
**Education level**			
Illiterate	2.25	1.29–3.94	0.005*
Up to 8 years	1.10	0.84–1.44	0.47
>8 years (ref.)			
**Marriage**			
Yes	0.71	0.54–0.94	0.02*
No (ref.)			
**Clinical form**			
Pleuro-pulmonary	0.90	0.68–1.18	0.48
Extra-pulmonary	1.05	0.68–1.6	0.62
Disseminated/Miliary (ref.)			
**Previous history of TB**			
Yes	0.53	0.37–0.74	0.0002*
No (ref.)			

* p<0.05

**Time from onset of symptoms to first visit to INI clinic.

**Table 6:** Multivariate survival analysis of factors associated health care delay** (n = 201).

Variable	HR	95% CI	p-value
**Education level**			
Illiterate	2.83	1.25–5.47	0.01*
Up to 8 years	1.17	0.78–1.74	0.44
>8 years (ref.)			
**Weight loss greater than 10%**			
Yes	0.54	0.37–0.80	0.006*
No (ref.)			
**Clinical form**			
Pleuro-pulmonary	1.06	0.70–1.63	0.27
Extra-pulmonary	1.21	0.66–2.21	0.16
Disseminated/Milliary (ref.)			
**Viral Load (Log10)**	0.37	0.24–0.54	0.02*

* p<0.05

** Time from first visit to clinic to initiation to initiation of TB therapy.

## Discussion

In this study, we found that illiteracy was associated with both longer patient delay and longer health care delay. In terms of patient delay, illiteracy may lead to inadequate knowledge of TB and therefore a low perception of risk and negative attitudes toward seeking care even in the presence of symptoms. [25] While we did not specifically examine these potential explanations, the association between illiteracy and patient delay is consistent with prior studies.[25] To the extent of our knowledge, this is the first time illiteracy is found to also be associated with longer health care delay. We believe this may be due to lower adherence to visits and laboratory tests requested by physicians during their initial diagnostic visit, possibly because of poor understanding of the medical investigation process and inaccurate perception of TB risk.

Surprisingly, low income was not associated with patient or health care delay. The fact that PLHIV in Rio de Janeiro have the right to free public transportation [26] could have reduced the impact of low income as a limitation to seeking care in response to TB symptoms and remaining linked to care during TB investigation.

Interestingly, being married and prior TB infection were inversely related to patient delay. Marital status may be a proxy for social support, which may lead to seeking care earlier. [25] Having had a previous episode of TB may lead to less delay because of greater knowledge and awareness of TB symptoms from the previous episode. Furthermore, it is possible that those who had a previous episode of TB were able to navigate the public health system more easily or get quicker access in the current episode.

In terms of health care delay, weight loss greater than 10% and higher viral load were clinical factors associated with less health care delay. These clinical indicators likely increase the physician’s suspicion of severe TB and lead to empiric treatment. The factors found to be associated with both patient and health care delay in this study contrast with those from the study performed by Coimbra et al. [15] where primarily clinical factors were found to be associated with greater total delay. Use of illicit drugs was the only social factor associated with later initiation of TB therapy in that study, but this finding was not replicated in our study. Furthermore, based on the study done by Coimbra et al. and others from different countries [15,25], we had expected to find that AFB smear and Xpert-MTB-RIF results were associated with health care delay. Interestingly, the results of microbiological testing did not impact delays in our study, and likely reflects the fact that empiric TB treatment is common in Brazil, as shown previously,[27] and may be more so in our sample as patients were seen by highly experienced infectious disease specialists. Although this finding may not be generalizable to primary care centers in Brazil, it is representative of the Brazilian scenario for HIV/TB co-infection, where symptomatic cases of HIV are investigated and treated empirically in specialized infectious disease centers.

The median patient and health care delay in this sample was longer than reported in many prior studies.[14] However, in most cases the populations are not comparable, as most studies are with HIV negative individuals with pulmonary TB. In contrast, we have included in our study PLHIV with pulmonary and extra-pulmonary TB that are potentially more difficult to diagnose. [18,28]

When looking at the delay times by HIV status we found that patient delay for individuals newly diagnosed with HIV was more than two times that of patients with previous diagnosis of HIV, although the variable “HIV status” was not significant in the model. This delay may result from a lower clinical suspicion of TB in the newly HIV positive group by both patients and health professionals. We considered the median patient delay time in those with remote diagnosis of HIV also excessive given that most were linked to HIV clinics and therefore are expected to have greater awareness regarding TB with easier access to health care in the event of new symptoms. The long delay in this group may result from low adherence to HIV care and medication non-adherence as suggested by the low number of individuals with viral suppression at the time of TB diagnosis. This raises the possibility of resistance to seek care as a common feature of this group, possibly driven by several factors such as stigma that were not investigated. Another possible explanation may be long wait times to schedule appointments, regardless of being linked to secondary centers. Health care delay was longest among individuals previously diagnosed with HIV. In our study, this could be explained by the fact that many newly diagnosed patients already arrive at INI clinic as a suspected case of TB, which may accelerate time to diagnosis.

Our study has several limitations. The first visit to INI may not have been the first health care center the patient presented to and thus the patient delay time may have been affected by health care aspects as well, as opposed to only patient related factors. In addition, the time from symptom onset was retrospectively self-reported and thus recall bias is possible. Furthemore, we classified TB forms as pleuropulmonary, extrapulmonary or disseminated/miliary rather than the Geneva classification for TB. Our classification approach was used in a previous study of the same population that found TB delay as a cause of mortality. [8] We believe that the classification used in our study did not impact the results, since our sample only included 2 patients with pleural TB that would be in a different group using the Geneva classification. Another limitation is that our study did not include other factors including: effect of seeking care during traditional clinical hours that overlap with patient work hours, stigma, knowledge of TB, culture of self-medication, access to care, effectiveness of the system in diagnosing TB, and time taken to refer patients to specialized centers. There is a need for more studies that include such factors. Furthermore, it is important that studies be done in other areas and settings in Brazil, as drivers of delayed treatment of TB may differ across the country.

Targeted efforts to reduce TB treatment delay in Brazil and elsewhere must concentrate on areas of high prevalence of TB among PLHIV. It is important to recognize vulnerable populations and specific factors associated with delay in order to develop focused interventions. Our findings broadly highlight the need to reduce inequalities and create more focused multidisciplinary interventions aimed at vulnerable groups. Illiteracy was found to affect both patient and health care delay. We have found a significant effect of iliteracy in spite of the number of illiterate individuals in the sample being small, suggesting that this finding represents a true effect. Furthermore, the confidence intervals on the estimate were not excessively wide, again suggesting the stability of the effect of iliteracy on treatment delay. The 6% prevalence of iliteracy in our sample corresponds with national estimates of approximately 8% iliteracy rate in individuals older than 15 years in 2015. [29] Adding to the illiteracy rate, a national survey done in 2009, found 20.3% individuals older than 15 years to be functional illiterates,[30] that is, unable to understand, evaluate, use and engage with written texts.[31] Another survey done in 2011 [32] found 27% functional illiteracy among 15 to 64 years old in Brazil. While policies aimed to reduce these statistics are needed and may result in significant reduction of TB delay in the long term, it is also important to consider that the efforts in education about TB may be failing to reach the illiterate population. One possible explanation is that the campaigns use mostly print based material. In the future, the inclusion of more audio-visual media such as campaigns on radio and television may be beneficial. In addition to more accessible campaigns for education about TB, the identification of illiterates and individuals with low social support at health facilities can be helpful if followed by careful orientation about the disease and how to navigate the health care system, closer follow-up, and active search when needed.

The identification of vulnerable populations across the country is important for the achievement of better outcomes and for reduction of TB-HIV co-infection. In this study, illiteracy was an important individual factor and the design of TB awareness interventions targeting this is a challenge that needs to be addressed. Furthermore, sustainable actions to reduce inequalities and improve education at the population level must be undertaken.

## Supporting information

S1 File De-identified dataset. Data set used for data analysis.
(XLSX)
