# Correction: Factors associated with tuberculosis treatment delay in patients co-infected with HIV in a high prevalence area in Brazil

**DOI:** 10.1371/journal.pone.0202292

**Published:** 2018-08-08

**Authors:** Betânia M. F. Nogueira, Valéria C. Rolla, Kevan M. Akrami, Susan M. Kiene

## Notice of Republication

An incorrect version of S1 File was published in error. This article was republished on July 27, 2018 to correct for this error. Please download this article again to view the correct version.
